# Supplementary material for: Modeling glioblastoma heterogeneity as a dynamic network of cell states
Source: Mol Syst Biol. 2021 Sep 16;17(9):e10105. doi: 10.15252/msb.202010105 (PMC8444284; doi:10.15252/msb.202010105)
Supplement: Supplementary file 6 — Source Data for Figure 5 [file MSB-17-e10105-s004.zip › Figure5A_sourcedata/GSEA_3017/nosigdb_stateA.GseaPreranked.1622795026485/GAROFANO_IAVARONE_GPM_SUBTYPE.html]

Details for gene set GAROFANO\_IAVARONE\_GPM\_SUBTYPE[GSEA]

|  || Dataset | state53017 |
| Phenotype | NoPhenotypeAvailable |
| Upregulated in class | na\_neg |
| GeneSet | GAROFANO\_IAVARONE\_GPM\_SUBTYPE |
| Enrichment Score (ES) | -0.42572224 |
| Normalized Enrichment Score (NES) | -2.851674 |
| Nominal p-value | 0.0 |
| FDR q-value | 0.0 |
| FWER p-Value | 0.0 |
Table: GSEA Results Summary

  

Fig 1: Enrichment plot: GAROFANO\_IAVARONE\_GPM\_SUBTYPE      
 Profile of the Running ES Score & Positions of GeneSet Members on the Rank Ordered List

  

| PROBE | GENE SYMBOL | GENE\_TITLE | RANK IN GENE LIST | RANK METRIC SCORE | RUNNING ES | CORE ENRICHMENT || 1 | GSTO1 |  |  | 15 | 0.718 | -0.0069 | No |
| 2 | IGFBP3 |  |  | 26 | 0.641 | -0.0091 | No |
| 3 | VIM |  |  | 46 | 0.543 | -0.0234 | No |
| 4 | ECHS1 |  |  | 67 | 0.487 | -0.0398 | No |
| 5 | SAR1A |  |  | 85 | 0.468 | -0.0529 | No |
| 6 | ADAM12 |  |  | 90 | 0.464 | -0.0506 | No |
| 7 | STC1 |  |  | 92 | 0.463 | -0.0448 | No |
| 8 | CTSC |  |  | 96 | 0.456 | -0.0415 | No |
| 9 | RGS10 |  |  | 110 | 0.445 | -0.0502 | No |
| 10 | SLC26A2 |  |  | 121 | 0.428 | -0.0555 | No |
| 11 | YME1L1 |  |  | 145 | 0.412 | -0.0766 | No |
| 12 | S100A4 |  |  | 148 | 0.409 | -0.0728 | No |
| 13 | DNAJC1 |  |  | 151 | 0.407 | -0.0690 | No |
| 14 | ANLN |  |  | 152 | 0.407 | -0.0629 | No |
| 15 | BAMBI |  |  | 175 | 0.391 | -0.0830 | No |
| 16 | MRPS6 |  |  | 181 | 0.389 | -0.0831 | No |
| 17 | PLS3 |  |  | 236 | 0.355 | -0.1417 | No |
| 18 | ITGB1 |  |  | 256 | 0.346 | -0.1590 | No |
| 19 | SDF2L1 |  |  | 257 | 0.345 | -0.1538 | No |
| 20 | HSPB1 |  |  | 312 | 0.322 | -0.2129 | No |
| 21 | TUBA1C |  |  | 322 | 0.318 | -0.2188 | No |
| 22 | CLIC1 |  |  | 342 | 0.311 | -0.2366 | No |
| 23 | PLAU |  |  | 347 | 0.310 | -0.2367 | No |
| 24 | CREB3L2 |  |  | 348 | 0.310 | -0.2320 | No |
| 25 | SLC20A1 |  |  | 361 | 0.306 | -0.2416 | No |
| 26 | TTF2 |  |  | 367 | 0.305 | -0.2429 | No |
| 27 | TXNDC5 |  |  | 406 | 0.291 | -0.2836 | No |
| 28 | ANXA7 |  |  | 408 | 0.291 | -0.2804 | No |
| 29 | P4HA1 |  |  | 411 | 0.290 | -0.2784 | No |
| 30 | GALNT7 |  |  | 417 | 0.289 | -0.2799 | No |
| 31 | CSGALNACT2 |  |  | 419 | 0.288 | -0.2768 | No |
| 32 | PFKP |  |  | 454 | 0.280 | -0.3128 | No |
| 33 | BCL3 |  |  | 455 | 0.280 | -0.3086 | No |
| 34 | ARMCX2 |  |  | 457 | 0.279 | -0.3056 | No |
| 35 | INPP5F |  |  | 469 | 0.276 | -0.3144 | No |
| 36 | MT2A |  |  | 473 | 0.275 | -0.3138 | No |
| 37 | REEP3 |  |  | 496 | 0.271 | -0.3358 | No |
| 38 | SH3BGRL3 |  |  | 512 | 0.269 | -0.3495 | No |
| 39 | EBF1 |  |  | 519 | 0.267 | -0.3526 | No |
| 40 | MANF |  |  | 521 | 0.266 | -0.3498 | No |
| 41 | PXDN |  |  | 524 | 0.266 | -0.3482 | No |
| 42 | NFE2L3 |  |  | 548 | 0.260 | -0.3715 | No |
| 43 | SEC23IP |  |  | 578 | 0.253 | -0.4020 | No |
| 44 | ITGAV |  |  | 599 | -0.250 | -0.4219 | Yes |
| 45 | PLEKHA5 |  |  | 600 | -0.252 | -0.4182 | Yes |
| 46 | CD99 |  |  | 604 | -0.252 | -0.4179 | Yes |
| 47 | SYPL1 |  |  | 608 | -0.255 | -0.4176 | Yes |
| 48 | ZFHX3 |  |  | 609 | -0.256 | -0.4137 | Yes |
| 49 | BST2 |  |  | 614 | -0.259 | -0.4146 | Yes |
| 50 | TACC1 |  |  | 623 | -0.260 | -0.4201 | Yes |
| 51 | IMMP2L |  |  | 626 | -0.261 | -0.4185 | Yes |
| 52 | MALT1 |  |  | 627 | -0.261 | -0.4146 | Yes |
| 53 | DPYD |  |  | 630 | -0.262 | -0.4130 | Yes |
| 54 | FLNA |  |  | 636 | -0.264 | -0.4150 | Yes |
| 55 | SHROOM3 |  |  | 639 | -0.264 | -0.4133 | Yes |
| 56 | GNG12 |  |  | 640 | -0.265 | -0.4093 | Yes |
| 57 | CNIH3 |  |  | 648 | -0.269 | -0.4136 | Yes |
| 58 | SOCS3 |  |  | 651 | -0.270 | -0.4119 | Yes |
| 59 | CHST2 |  |  | 652 | -0.271 | -0.4078 | Yes |
| 60 | IRS1 |  |  | 657 | -0.273 | -0.4084 | Yes |
| 61 | ZNF385D |  |  | 658 | -0.273 | -0.4043 | Yes |
| 62 | GCC2 |  |  | 661 | -0.276 | -0.4025 | Yes |
| 63 | PGM2L1 |  |  | 662 | -0.276 | -0.3983 | Yes |
| 64 | SEZ6L2 |  |  | 672 | -0.283 | -0.4047 | Yes |
| 65 | EFEMP2 |  |  | 686 | -0.294 | -0.4157 | Yes |
| 66 | GPC4 |  |  | 687 | -0.295 | -0.4112 | Yes |
| 67 | AKAP12 |  |  | 690 | -0.296 | -0.4091 | Yes |
| 68 | ATP6AP2 |  |  | 705 | -0.304 | -0.4211 | Yes |
| 69 | MDFIC |  |  | 709 | -0.308 | -0.4200 | Yes |
| 70 | DSTN |  |  | 710 | -0.309 | -0.4154 | Yes |
| 71 | NAV3 |  |  | 713 | -0.311 | -0.4130 | Yes |
| 72 | NME3 |  |  | 715 | -0.311 | -0.4095 | Yes |
| 73 | S100A6 |  |  | 717 | -0.313 | -0.4060 | Yes |
| 74 | FZD7 |  |  | 721 | -0.316 | -0.4048 | Yes |
| 75 | EMP1 |  |  | 724 | -0.322 | -0.4023 | Yes |
| 76 | PRKAR2B |  |  | 726 | -0.323 | -0.3986 | Yes |
| 77 | RFFL |  |  | 728 | -0.323 | -0.3949 | Yes |
| 78 | CBR1 |  |  | 732 | -0.327 | -0.3936 | Yes |
| 79 | TRIM56 |  |  | 748 | -0.341 | -0.4062 | Yes |
| 80 | LPCAT2 |  |  | 765 | -0.358 | -0.4197 | Yes |
| 81 | BOC |  |  | 766 | -0.359 | -0.4143 | Yes |
| 82 | TBX2 |  |  | 768 | -0.359 | -0.4101 | Yes |
| 83 | PSMB8 |  |  | 772 | -0.362 | -0.4082 | Yes |
| 84 | IGFBP7 |  |  | 780 | -0.368 | -0.4109 | Yes |
| 85 | SPARC |  |  | 782 | -0.369 | -0.4065 | Yes |
| 86 | TPM1 |  |  | 784 | -0.369 | -0.4022 | Yes |
| 87 | BDNF |  |  | 790 | -0.374 | -0.4024 | Yes |
| 88 | DCBLD2 |  |  | 791 | -0.375 | -0.3968 | Yes |
| 89 | CHPF |  |  | 793 | -0.375 | -0.3923 | Yes |
| 90 | SQSTM1 |  |  | 795 | -0.379 | -0.3878 | Yes |
| 91 | TRAM2 |  |  | 797 | -0.382 | -0.3832 | Yes |
| 92 | NPC2 |  |  | 800 | -0.383 | -0.3798 | Yes |
| 93 | CRIM1 |  |  | 805 | -0.388 | -0.3787 | Yes |
| 94 | SSX2IP |  |  | 810 | -0.392 | -0.3775 | Yes |
| 95 | MRC2 |  |  | 813 | -0.395 | -0.3739 | Yes |
| 96 | LTBP1 |  |  | 815 | -0.397 | -0.3691 | Yes |
| 97 | FTL |  |  | 825 | -0.409 | -0.3736 | Yes |
| 98 | PRRX1 |  |  | 829 | -0.413 | -0.3709 | Yes |
| 99 | CD59 |  |  | 831 | -0.422 | -0.3658 | Yes |
| 100 | PAM |  |  | 832 | -0.422 | -0.3594 | Yes |
| 101 | PLOD2 |  |  | 838 | -0.431 | -0.3588 | Yes |
| 102 | TGFB2 |  |  | 840 | -0.432 | -0.3535 | Yes |
| 103 | MAP1LC3A |  |  | 842 | -0.436 | -0.3481 | Yes |
| 104 | CHPT1 |  |  | 844 | -0.437 | -0.3427 | Yes |
| 105 | SERPINB6 |  |  | 861 | -0.452 | -0.3548 | Yes |
| 106 | IFITM2 |  |  | 864 | -0.460 | -0.3502 | Yes |
| 107 | UPP1 |  |  | 867 | -0.467 | -0.3456 | Yes |
| 108 | ANXA2 |  |  | 872 | -0.482 | -0.3430 | Yes |
| 109 | PRSS23 |  |  | 874 | -0.486 | -0.3369 | Yes |
| 110 | PMEPA1 |  |  | 877 | -0.497 | -0.3318 | Yes |
| 111 | MOXD1 |  |  | 878 | -0.498 | -0.3242 | Yes |
| 112 | NRCAM |  |  | 887 | -0.526 | -0.3258 | Yes |
| 113 | CALD1 |  |  | 889 | -0.527 | -0.3190 | Yes |
| 114 | PDP1 |  |  | 891 | -0.531 | -0.3122 | Yes |
| 115 | A2M |  |  | 898 | -0.545 | -0.3111 | Yes |
| 116 | ARHGAP29 |  |  | 899 | -0.548 | -0.3028 | Yes |
| 117 | PLK2 |  |  | 901 | -0.551 | -0.2957 | Yes |
| 118 | ITGB5 |  |  | 903 | -0.558 | -0.2884 | Yes |
| 119 | SDC2 |  |  | 908 | -0.568 | -0.2846 | Yes |
| 120 | SAT1 |  |  | 909 | -0.573 | -0.2760 | Yes |
| 121 | DKK3 |  |  | 912 | -0.579 | -0.2696 | Yes |
| 122 | ARID5B |  |  | 915 | -0.586 | -0.2631 | Yes |
| 123 | NEDD9 |  |  | 923 | -0.625 | -0.2620 | Yes |
| 124 | FRMD4B |  |  | 925 | -0.626 | -0.2537 | Yes |
| 125 | SPP1 |  |  | 927 | -0.630 | -0.2454 | Yes |
| 126 | CEBPD |  |  | 939 | -0.690 | -0.2480 | Yes |
| 127 | LIMCH1 |  |  | 944 | -0.716 | -0.2420 | Yes |
| 128 | SERPINE1 |  |  | 951 | -0.774 | -0.2374 | Yes |
| 129 | MMP2 |  |  | 953 | -0.829 | -0.2261 | Yes |
| 130 | IFITM3 |  |  | 954 | -0.830 | -0.2135 | Yes |
| 131 | CD44 |  |  | 957 | -0.851 | -0.2031 | Yes |
| 132 | COL5A2 |  |  | 958 | -0.856 | -0.1901 | Yes |
| 133 | RND3 |  |  | 959 | -0.864 | -0.1771 | Yes |
| 134 | CSRP2 |  |  | 960 | -0.877 | -0.1639 | Yes |
| 135 | CNN3 |  |  | 961 | -0.883 | -0.1505 | Yes |
| 136 | TAGLN2 |  |  | 963 | -0.891 | -0.1383 | Yes |
| 137 | CRYAB |  |  | 967 | -0.940 | -0.1276 | Yes |
| 138 | LGALS3 |  |  | 970 | -0.989 | -0.1151 | Yes |
| 139 | ANXA1 |  |  | 972 | -1.018 | -0.1009 | Yes |
| 140 | CTGF |  |  | 976 | -1.182 | -0.0866 | Yes |
| 141 | CYR61 |  |  | 978 | -1.296 | -0.0683 | Yes |
| 142 | PMP22 |  |  | 982 | -1.519 | -0.0489 | Yes |
| 143 | IGFBP5 |  |  | 983 | -1.595 | -0.0248 | Yes |
| 144 | CCL2 |  |  | 985 | -1.881 | 0.0024 | Yes |
Table: GSEA details [plain text format]

  

Fig 2: GAROFANO\_IAVARONE\_GPM\_SUBTYPE: Random ES distribution      
 Gene set null distribution of ES for **GAROFANO\_IAVARONE\_GPM\_SUBTYPE**
